# Bioavailability and leaching of Cd and Pb from contaminated soil amended with different sizes of biochar

**DOI:** 10.1098/rsos.181328

**Published:** 2018-11-21

**Authors:** Alaa Hasan Fahmi, Abd Wahid Samsuri, Hamdan Jol, Daljit Singh

**Affiliations:** 1 Department of Land Management, Faculty of Agriculture, Universiti Putra Malaysia, 43400 Serdang, Selangor, Malaysia; 2 Department of Soil Science and Water Resources, College of Agriculture, University of Diyala, Diyala, Iraq

**Keywords:** biochar particle size, contaminated soil, heavy metals, bioavailability, leaching

## Abstract

Biochars have been successfully used to reduce bioavailability and leaching of heavy metals in contaminated soils. The efficiency of biochar to immobilize heavy metals can be increased by reducing the particle size, which can increase the surface area and the cation exchange capacity (CEC). In this study, the empty fruit bunch biochar (EFBB) of oil palm was separated into two particle sizes, namely, fine (F-EFBB < 50 µm) and coarse (C-EFBB > 2 mm), to treat the contaminated soil with Cd and Pb. Results revealed that the addition of C-EFBB and F-EFBB increased the pH, electrical conductivity and CEC of the contaminated soil. The amounts of synthetic rainwater extractable and leachable Cd and Pb significantly decreased with the EFBB application. The lowest extractable and leachable Cd and Pb were observed from 1% F-EFBB-treated soil. The amount of extractable and leachable Cd and Pb decreased with increasing incubation times and leaching cycles. The application of F-EFBB to Cd and Pb-contaminated soil can immobilize the heavy metals more than that of C-EFBB. Therefore, the EFBB can be recommended for the remediation of heavy metal-contaminated soils, and a finer particle size can be applied at a lower application rate than the coarser biochar to achieve these goals.

## 1. Introduction

As Cd and Pb are heavy metals that are non-biodegradable in the soil, they exhibit long-term resistance in the environment and become toxic to humans, plants and animals [1,2]. These metals are capable of moving in a soil solution or along the surface water and leaching into the underground water because of their dissolution from soil minerals by acidic water or through industrial discharge, mining waste and landfill [3,4]. Therefore, to reduce their mobility and bioavailability, thus immobilizing them in the soil and in the environment, sorbent materials and precipitating agents are usually employed to achieve these goals [1,4,5].

As an adsorbent, biochar has the ability to immobilize heavy metals in the soil and in the environment and to increase the overall productivity of the soil [6]. Biochar is an environment-friendly and cost-effective sorbent made from plant and animal materials [4]. The ability of biochar to alleviate organic and inorganic compounds is attributed to its physico-chemical properties, which include a porous structure, active functional groups, an extended specific surface area, a cation exchange capacity (CEC) and a great organic carbon content [7,8]. Various studies revealed that the immobilization of heavy metals in soil is ascribed to the field application of biochar, which assists in reducing bioavailability and leaching through adsorption and chemical transformation, thus helping to control the toxicity of these heavy metals in the soil and in the environment [9–11]. The application of empty fruit bunch biochar (EFBB) significantly decreased heavy metal leaching [12]. According to this study, tailings soil treated with EFBB had a significant decrease in the mobility of Pb, Mn and Cu, and this decrease was attributed to the physico-chemical properties of the biochar, most importantly, the surface area and functional groups.

Biochar produced at a low temperature is characterized by highly functional groups and low homogeneity and adsorption capacity [13]. To increase the increased surface area and adsorption capacity of biochar, several studies suggested crushing it to a lower particle size [14,15]. In our previous sorption study on an aqueous solution (Fahmi *et al.*, unpublished data), using EFBB produced with low temperature (250°C), we found that the biochar with a particle size of less than 50 µm had a higher adsorption capacity for Cd and Pb than the biochar with a larger particle size due to the exposure of the inner pores and their functional groups. Therefore, the application of this biochar with a small particle size was hypothesized to help in ameliorating bioavailability and leaching of Cd and Pb in the soil. This is one of the limited studies on the effect of different particle sizes and application rates on the bioavailability and leaching of heavy metals. Shen *et al.* [16] found that biochar had no significant effect on the immobilization of Pb in contaminated soil. Nevertheless, they used different particle sizes of biochar (less than or equal to 0.15 mm and less than or equal to 2 mm) with a 1% application rate produced at 600°C. The authors attributed the result to the characterization of the soil, which contained kaolin (clay mineral), and it was difficult for biochar to precipitate Pb under the acidic environment. Then, they concluded that the biochars failed to treat clay-rich soils contaminated with heavy metals. Therefore, it is important to estimate the ability of biochar with higher functional groups content (pyrolysis at low temperature less than 250°C) as well as the effort of maximizing the exposure of these functional groups by crushing the biochar to less than 50 µm on the bioavailability and leaching of Cd and Pb in soil contenting kaolinite mineral and rich in clay.

This study was conducted to determine the effect of two contrasting EFBB particle sizes, namely, fine (F-EFBB < 50 µm) and coarse (C-EFBB > 2 mm), at different rates (0%, 0.5% and 1%) on the bioavailability and leaching of Cd and Pb in acidic Malaysian soil.

## 2. Material and methods

### 2.1. Chemicals and reagents

All the chemical reagents were of analytical grade, and the solutions were prepared using Milli-Q system (Direct-Q^®^ 3 UV) ultrapure water (electrical resistivity 18.2 MΩ cm^−1^). Analytical grade lead (II) nitrate with 99.99% purity and cadmium (II) nitrate tetrahydrate with 98.00% purity were purchased from Sigma-Aldrich (USA). Barium chloride (98.00% purity), sodium bicarbonate (greater than 99.70% purity), sodium carbonate (99.90% purity), sodium hydroxide (purity 99.00%), hydrochloric acid (37.00 % ACS grade), nitric acid (65.00% GR grade) and phenolphthalein (99.00% ACS grade) were purchased from Merck (Germany).

### 2.2. Soil sampling and spiking process

Munchong series soil was sampled from the Ladang 10 of the Faculty of Agriculture, Universiti Putra Malaysia (UPM). The global positioning system points of the sampling site were 02.98787 north and 101.71372 east. The soil samples were taken from a 0–15 cm depth using an auger and a shovel. The samples were transported to the drying room of the Department of Land Management, Faculty of Agriculture, UPM, where they were air-dried and ground using mortar and pestle and sieved with a 2 mm sieve. The soil was spiked with Cd and Pb (12 and 500 mg kg^−1^, respectively). The chemical compounds used to spike the soil were Pb(NO_3_)_2_ and Cd(NO_3_)_2_. These Cd and Pb compounds were dissolved in Millipore water to reach the desired concentration for the target level and then transferred to the soil, which was mixed homogeneously. The soil was then incubated for 60 days at room temperature (25 ± 2 °C) at 60% field capacity, which was maintained throughout the duration. After the incubation period, the soil was sieved with a 2 mm stainless steel sieve to obtain homogeneous samples. Then, the sample was stored at room temperature prior to use.

### 2.3. Physico-chemical analysis of soil

The texture and the particle size distribution of the Munchong soil were determined by the pipette method as described by Teh & Talib [17]. The separated clay fraction from the soil texture and the particle size analysis was collected to determine the soil mineralogy. About 1 ml of the suspended clay was spread on the microscopic glass slides. These slides were left to dry and then sent for X-ray diffraction analysis by a Phillips PW 3440/60 X'pert Pro X-Ray diffractometer (XRD) using a CuK-alpha radiation target operated at 40 kV and 30 mA. The pH and electrical conductivity (EC) were measured in triplicate samples of the soil using a Metrohm 827 pH meter and a Eutech Instruments CON 700 EC meter, respectively. The loss-on-ignition method was used to determine the soil organic matter content in the contaminated soil [18].

The percentages of the total C, N and S contents in the soil were determined from the oven-dried samples (105°C). To prepare the samples, the soil was sieved by passing it through a 0.5 mm sieve. Approximately 0.1 g of the soil was weighed into a clean, carbon-free combustion boat and combusted at 1350°C in an oxygen atmosphere using a LECO CNS erumace analyser.

The CEC of the soil sample was determined using the method established by Rengasamy & Churchman [19]. Briefly, 2 g of soil was weighed into a plastic vial, and 20 ml of 1 M ammonium acetate (NH_4_OAc) buffered at pH 7 was added. The mixture was shaken on a mechanical shaker for 20 min. Then, the soil was left to settle, and the suspension was pipetted. A total of 20 ml of alcohol was added and shaken for 20 min, and the suspension was allowed to settle and pipetted. The addition of 20 ml of alcohol was repeated. Then, 20 ml of 1 M K_2_SO_4_ was added, the sample was shaken for 20 min and the suspension was allowed to settle and pipetted. The extracted ammonium by 1 M K_2_SO_4_ was used to calculate the CEC in the soil by determining the amount of NH_4_^+^ using the autoanalyser.

The total elemental content of the soil samples was extracted using the aqua regia digestion method [20]. In summary, 0.5 g samples were placed in a 100 ml Pyrex digestion tube, and 6 ml of 35% HCl and 2 ml of 69% HNO_3_ were added to initiate the digestion (110°C). After cooling, 10 ml 1.2% HNO_3_ was added, and the mixture was reheated (80°C) for 30 min and allowed to cool. The mixture was made to reach 20 ml with deionized water. The suspension was filtered through an ashless Whatman 42 filter and stored in polyethylene bottles at 4°C for further analyses. The nutrients were determined using the PerkinElmer Optima 8300 ICP-OES.

### 2.4. Preparation and characterization of biochar samples

The EFBB was purchased from the Malaysian Palm Oil Board (MPOB) in Bangi Lama, Selangor, Malaysia. The biochar was prepared using a low temperature (250°C) slow pyrolysis process in a horizontal rotary kiln. The rotating motion of the kiln moves the EFB biomass along the kiln and the speed is controlled based on the period of the heating required. The biochar was brought to the laboratory, where it was passed through a metal sieve (Laboratory Test Sieve Endecotts Ltd., UK) to separate them into coarse EFBB (greater than 2 mm; C-EFBB). The F-EFBB (less than 50 µm) was achieved by milling the biochar with a planetary milling machine (Pulverisette 4 Vario-Planetary Mill) set to 1200 r.p.m. for 3 h. Both biochar samples (C-EFBB and F-EFBB) were kept at room temperature prior to analysis.

The surface areas of the C-EFBB and F-EFBB were determined by N_2_ adsorption at 77 K, and the Brunauer–Emmett–Teller technique was employed using the Autosorb-1 surface area analyser (Quantochrome Instruments, USA). Prior to the N_2_ analysis, the adsorbent samples were degassed at 200°C for 9 h. The multipoint BET method was used to calculate the total surface area.

The pH values of the C-EFBB and F-EFBB were measured according to Savova *et al.* [21] using a pH meter (Model Metrohm^®^ 827, USA). The EC measurements of the C-EFBB and F-EFBB were measured by soaking the sample in Millipore water at a solid/water ratio of 1 : 5 (w/v) and agitated for 24 h using a CON 700 EC meter (Eutech Instruments, USA).

The CEC of the C-EFBB and F-EFBB was determined by a compulsive exchange method according to [22] as simplified by Shen *et al.* [15]. About 1 g of the C-EFBB and F-EFBB were weighed into the Falcon centrifuge tubes, and 20 ml of the 0.5 M BaCl_2_ was added. The resultant mixture was agitated at 200 r.p.m. for 2 h and filtered using a 0.45 µm filter. The concentration of exchangeable bases, such as Na, Mg, K and Ca aside from Mn and Fe in the filtrate, was quantified by atomic adsorption spectrometry (AAnalyst 400, PerkinElmer, USA). Further, Al was subsequently diluted and acidified and then quantified by an inductively coupled plasma–optical emission spectrometer (ICP-OES, Perkin Elmer Optima 8300 DV, USA). The CEC was calculated as the sum of all cations.

C and N in the EFBB were determined using a TruSpec CHNS analyser (Leco^®^, USA). The concentrations of heavy metals in the EFBB were determined according to the method of McGrath & Cunliffe [20]. Briefly, the air-dried samples were ground to achieve a powder particle size. Thereafter, 0.25 mg of the sample treatments was weighed and transferred to digestion vessels. Then, 6 ml HCl and 2 ml HNO_3_ were added to the samples and digested at 110°C. The digestate was cooled, and then 10 ml of 1.2% HNO_3_ was added. The digestate was reheated to 80°C for 30 min and then made to reach 20 ml with Millipore water. The samples were vortexed and filtered through a Whatman No. 42 filter paper into plastic vials. The samples were analysed for their total elemental content using the ICP-OES.

The zeta potentials of the C-EFBB and F-EFBB were determined by weighing a 0.02 g sample into a 250 ml conical flask containing 100 ml of 0.1 M NaCl solution. The pH of the resultant suspension was adjusted at intervals of pH 2–9 using either 0.025 M HCl or 0.025 NaOH. A bath-type sonicator set to 40 kHz and equipped with a 300 W power supply line for 120 min at 30°C was used to disperse the suspension ultrasonically. The suspension was then left at room temperature for 24 h. Thereafter, the electrophoresis mobility measurement was conducted using a nanoparticle and zeta potential system (Model: Zetasizer Nano ZS), and the data were analysed using the Zeta Sizer Nano software v. 7.1.

### 2.5. Incubation study

The incubation study was conducted in the General Research Laboratory of the Department of Land Management, Faculty of Agriculture, UPM, at room temperature (25 ± 2°C) using a container 11 cm in length and 9 cm in diameter. The C-EFBB or F-EFBB was mixed with 100 g of contaminated soil with Cd and Pb at 0%, 0.5% or 1% (w/w). Each experimental unit was set up in triplicate. The experimental units were arranged in a randomized complete block design (RCBD) and incubated for 60 days at 60% field capacity. The containers were covered with parafilm with small holes poked into them for aeration. After the end of the incubation period, the pH, EC and CEC of each experimental unit were measured as described previously.

### 2.6. Bioavailability study

This study was conducted to examine the effect of the different particle sizes of EFBBs (C-EFBB and F-EFBB) and the different application rates (0%, 0.5% or 1%) on the bioavailability of Pb and Cd in soil contaminated with Cd and Pb at different incubation times (0, 1, 2, 3, 4, 6 and 8 weeks). About 20 g of contaminated soil was weighed into the Falcon tubes. Then, the EFBBs were added at a specific rate and mixed with the soil to achieve homogeneous mixtures. Each experimental unit was set up in triplicate. The Falcon tubes were then sealed with parafilm, and small holes were poked into them to allow for aeration. The tubes were incubated in the General Research Laboratory of the Department of Land Management, Faculty of Agriculture, UPM, at room temperature (25 ± 2°C), with the moisture content maintained throughout the entire study at 60% field capacity. The experimental units were arranged in an RCBD. At the end of each incubation period, 40 ml of synthetic rainwater (SRW) was added to the Falcon tubes, which were shaken on the end-to-end shaker for 24 h at 40 rpm. The SRW was prepared by adding 10 mM of sulfuric acid to the Milli-Q^®^ water dropwise until pH 4.5 was achieved [23]. The SRW was used to simulate the extraction of bioavailable Cd and Pb by rainwater. The filtrates were then acidified with 4% HNO_3_ (v/v) and kept in the fridge prior to the analysis of Cd and Pb by the ICP-OES.

### 2.7. Leaching study

The leaching study was conducted in the General Research Laboratory of the Department of Land Management, Faculty of Agriculture, UPM, at room temperature (25 ± 2°C) using leaching columns 30 cm in length and 5 cm in diameter. The bottoms of the leaching columns were plugged with cotton to prevent the soil from escaping. About 100 g of the contaminated soil was mixed with either C-EFBB or F-EFBB at 0%, 0.5% or 1% (w : w). Each experimental unit was triplicated. The soil mixture was placed into the leaching column, and a 10 cm headspace was left to add the SRW. The SRW is widely used to simulate contaminant leaching via rainfall [23–26], thus, it was used to leach the Cd and Pb from the contaminated soils in the current study. The treatments were arranged in an RCBD and incubated for 60 days at 60% field capacity. The tops of the leaching columns were sealed with parafilm, and small holes were poked into them to allow for aeration. At the end of 60 days, the columns were leached with 50 ml SRW for six cycles, and the leachates were collected and acidified with 4% HNO_3_ solution. The concentrations of Cd and Pb in the leachates were analysed using the ICP-OES as previously described.

### 2.8. Statistical analysis

All data were checked for normality and homogeneity of variances prior to statistical analysis. The precision of the data was calculated and expressed as a standard error (s.e.). Then, the data were subjected to an ANOVA procedure using the SAS software pack (version 9.4). The significance level was set to a 95% confidence level (*α* = 0.05), and the Tukey test was employed to test the significant differences among the treatment means.

## 3. Results

### 3.1. Soil and biochar properties

**Figure 1.**
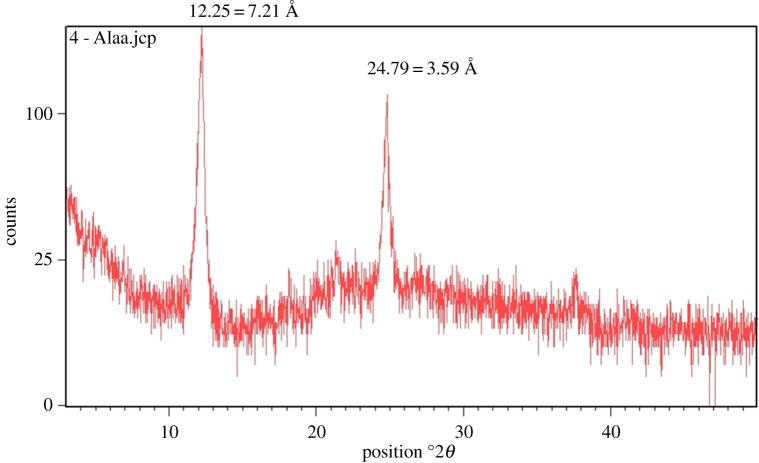
X-ray diffractograms of Munchong soil.

The XRD diffractogram pattern of the soil clay fraction indicated that kaolinite (peaks at 7.2 and 3.59) was the most abundant mineral in the soil sample (figure 1). The peaks on the XRD diffractograms were interpreted by the methods of Shamshuddin [27]. The presence of this mineral in the soil being studied was consistent with that found in the previous study by Shamshuddin & Fauziah [28]. The physiochemical properties of the soil are summarized in table 1.

**Table 1.** The physicochemical properties of soil. n.d. = not detected. The values are reported as the mean of triplicate samples with the corresponding standard error (s.e.).

parameters	value
pH H_2_O	4.54 ± 0.06
EC (ds m^−1^)	147.4 ± 1.50
CEC (cmol_+_ kg^−1^)	5.412 ± 0.05
OM (%)	3.691 ± 0.10
C (%)	0.864 ± 0.09
N (%)	0.072 ± 0.04
S (%)	0.091 ± 0.00
Fe (%)	0.841 ± 0.01
K (%)	0.088 ± 0.01
Al (%)	9.032 ± 0.16
Ca (mg kg^−1^)	n.d.
Mg (mg kg^−1^)	23 ± 0.2
Na (mg kg^−1^)	43.2 ± 0.58
Cu (mg kg^−1^)	17.2 ± 1.00
Mn (mg kg^−1^)	57.667 ± 3.92
Zn (mg kg^−1^)	60 ± 5.24
P (mg kg^−1^)	73 ± 7.91
clay (%)	62.00
silt (%)	6.40
sand (%)	31.83
texture (USDA)	clay

**Figure 2.**
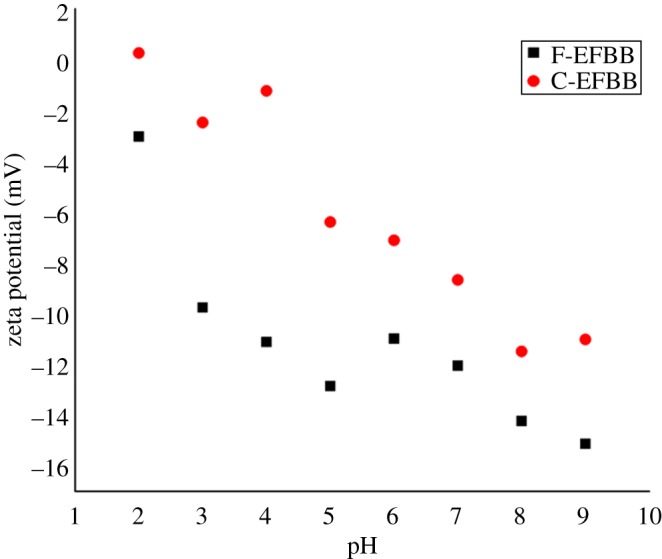
The zeta potential as a function of pH for F-EFBB and C-EFBB.

Figure 2 shows the point of zero charge (PZC) varying among the different particle sizes. At pH 2, the zeta potentials of the adsorbents were –3.03 and 0.257 mV for F-EFBB and C-EFBB, respectively. The values became more negative with increasing pH values. The PZC was not detected for the F-EFBB, thus implying that the surfaces were all negatively charged throughout all the pH ranges tested. This result is consistent with the greatest CEC recorded by the F-EFBB, as shown in table 2. The presence of oxygen-containing functional groups is most likely responsible for the higher surface negative charges [29]. Samsuri *et al.* [30] measured the zeta potential for EFBB and rice husk and did not detect the PZC for both biochars and the surface was negatively charged within the pH range. However, Yang *et al.* [31] found the PZC was 2.3 for water hyacinths biochar produced at 450°C. Table 2 shows the physico-chemical properties of the biochar.

**Table 2.** Physicochemical properties of the biochar.

parameters	C-EFBB	F-EFBB
surface area (m^2^ g^−1^)	0.90	2.57
pH	9.333 ± 0.035	9.603 ± 0.025
EC (dS m^−1^)	8.250 ± 0.02	11.407 ± 0.150
total C (%)	61.817 ± 0.511	61.817 ± 0.511
N (%)	1.096 ± 0.011	1.096 ± 0.011
CEC cmol_(+)_kg^−1^ by (BaCl_2_)	1.842 ± 0.506	16.464 ± 0.280

Table 3 shows the heavy metal concentrations in the EFBB and the IBI guideline values for heavy metals in biochar for soil application. In this study, none of the metals in the EFBB exceeded the IBI [32] guideline values.

**Table 3.** Risk assessment of heavy metal content in EFBB in comparison with IBI guidelines. The values are reported as the mean of triplicate samples with the corresponding standard error (s.e.).

heavy metal (mg kg^−1^)	EFBB	IBI limit	remarks
Mn	68.133 ± 0.266	—	—
Zn	94.933 ± 1.425	416–7400	passed
Cu	21.266 ± 0.581	143–6000	passed
Pb	2.733 ± 1.642	121–300	passed
Cd	2.066 ± 1.068	1.43–39	passed

### 3.2. Incubation study

Table 4 shows the pH, EC and CEC of the contaminated soil amended with EFBB of different particle sizes and rates after 60 days of incubation time. The soil treated with 1% of either C-EFBB or F-EFBB had higher pH than the same EFBB applied at 0.5%. The pH levels of all EFBB-treated soil were higher than the pH of the control soil. There was no significant pH difference between soil treated with different sizes of EFBB either at the 1% or at the 0.5% application rate. The lowest pH (4.237) was recorded in the control soil, whereas the highest values (4.630 and 4.667) were recorded in the 1% F-EFBB- and 1% C-EFBB-treated soil, respectively.

**Table 4.** The pH, EC, CEC value of contaminated soil amended with different sizes and rates of EFBB after 60 days of incubation time. Different letters show significant differences (*p* < 0.05) between the values in the same column.

biochar size	biochar rate	pH	EC (ds m^−1^)	CEC (cmol_+_ kg^−1^)
control	0%	4.237 ± 0.012^b^	538.000 ± 16.258^c^	5.41 ± 0.058^c^
C-EFBB	0.50%	4.330 ± 0.006^b^	667.333 ± 2.028^b^	5.72 ± 0.073^b^
	1%	4.667 ± 0.027^a^	810.333 ± 14.836^a^	7.50 ± 0.041^a^
F-EFBB	0.50%	4.343 ± 0.070^b^	691.333 ± 9.528^b^	5.54 ± 0.044^bc^
	1%	4.630 ± 0.078^a^	868.667 ± 25.983^a^	7.71 ± 0.000^a^

The EC values of soil treated with 1% C-EFBB or F-EFBB were significantly higher than those of soil treated with a 0.5% rate, and all EFBB-treated soil had higher EC values than the control. No effect of EFBB particle size was observed on the soil EC values either at the 0.5% or at the 1% application rate. The lowest EC value (538.000 ds m^−1^) was recorded by the control soil, whereas the highest EC values (810.333 ds m^−1^ and 868.667 ds m^−1^) were recorded by the 1% C-EFBB and 1% F-EFBB treatment, respectively.

The CEC values of the soil treated with 1% C-EFBB were significantly higher than those of the soil treated with 0.5% C-EFBB and the control, but the CEC value of 0.5% C-EFBB was not significantly different compared with the control. A similar trend was observed for the F-EFBB-treated soil. No effect of EFBB particle size was found on the soil CEC values either at the 0.5% or at the 1% application rate. The lowest CEC (5.41 cmol_+_ kg^−1^) was recorded in the control treatment, whereas the highest CEC values (7.50 cmol_+_ kg^−1^ and 7.7 cmol_+_ kg^−1^) were recorded in the 1% C-EFBB and F-EFBB treatment, respectively.

### 3.3. Bioavailability study

**Figure 3.**
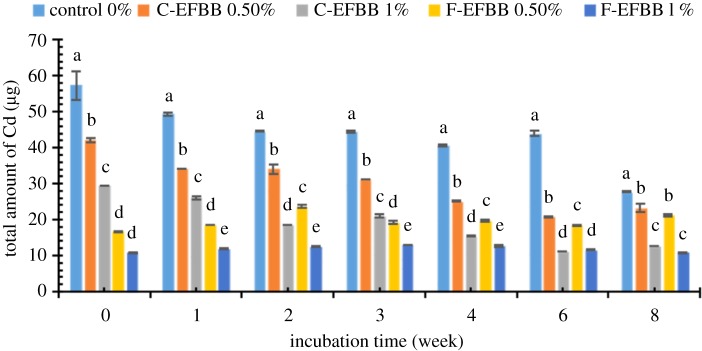
The amounts of Cd extracted with SRW from contaminated soil amended with different particle sizes and rates of biochar at different incubation time. (Different letters show significant differences (*p* < 0.05) between the treatment means in the same incubation time.)

**Figure 4.**
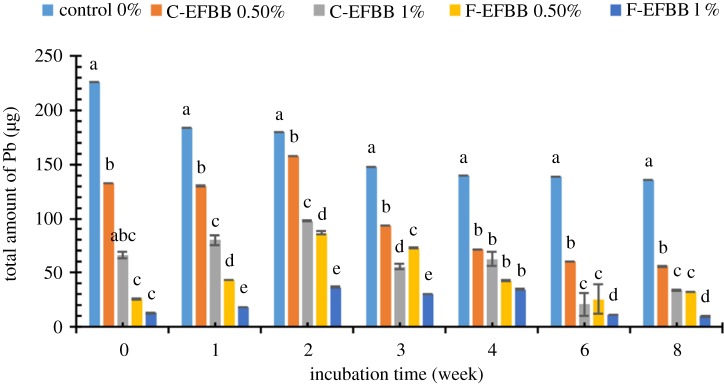
The amounts of Pb extracted with SRW from contaminated soil amended with different particle sizes and rates of biochar at different incubation time. (Different letters show significant differences (*p* < 0.05) between the treatment means in the same incubation time.)

The amounts of SRW-extractable Cd and Pb from the contaminated soil amended with the different particle sizes and rates of biochar incubated at different incubation times are presented in figures 3 and 4, respectively. Generally, the amounts of SRW-extractable Cd and Pb significantly decreased with the increasing incubation time. Figure 3 shows the amount of SRW-extractable Cd from soil amended with different particle sizes and rates of biochar over eight weeks. The application rate of 1% had a significantly lower amount of Cd extracted than that of 0.5%, and both their Cd amounts were lower than that of the control over incubation times for both EFBBs. The F-EFBB-treated soil had a lower amount of extractable Cd than the C-EFBB-treated soil at similar application rates. The lowest amounts of extracted Cd (10.786 µg and 12.680 µg) were recorded in soil applied with 1% F-EFBB and 1% C-EFBB, respectively, in eight weeks. The highest amount of extracted Cd (57.253 µg) was recorded in soil incubated with the control treatment for zero weeks.

Figure 4 shows the amount of SRW-extractable Pb from the soil amended with different particle sizes and rates of biochar for over eight weeks. The application rate of 1% had a significantly lower amount of extractable Pb than that of 0.5%, and both soils had a lower amount of extractable Pb than the control throughout the incubation weeks except on week 4, when no significant difference was found between the 1% and 0.5% application rates. F-EFBB had a significantly lower amount of extractable Pb than C-EFBB throughout the incubation weeks except on week 4 with a similar application rate. The lowest amount of extractable Pb (9.770 µg) was recorded with the 1% F-EFBB treatment in eight weeks, whereas the highest amount of extractable Pb (225.720 µg) was recorded with the control treatment on week 0.

### 3.4. Leaching study

Tables 5 and 6 show the amount of SRW-leachable Cd and Pb, respectively, from the contaminated soil amended with different particle sizes and rates of biochar over a number of leaching cycles. In general, the amounts of Cd and Pb leached from the contaminated soil were affected by the biochar particle size and application rate. The amounts of SRW-leachable Cd and Pb significantly decreased with the increasing leaching cycle. The leachates from leaching cycle number 5 had significantly lower amounts of Cd and Pb, whereas leaching cycle number 1 had significantly higher amounts.

**Table 5.** Amount of Cd (µg) in the leachate of contaminated soil amended with EFBB over leaching cycles. Means with the same small letter in the same column are not significantly different. Means with the same capital letter in the same row are not significantly different.

		amount of leachable Cd (µg) in different leachates					
size	rate	1	2	3	4	5	6
control	0%	173.27 ± 2.15^aA^	87.92 ± 0.79^aB^	12.08 ± 0.16^aC^	4.65 ± 0.00^aD^	0.40 ± 0.03^aD^	3.05 ± 1.28^abD^
C-EFBB	0.5%	116.95 ± 0.60^bA^	48.15 ± 0.56^bB^	4.48 ± 0.07^cC^	0.12 ± 0.04^cD^	0.150 ± 0.15^abD^	4.83 ± 1.66^aC^
	1%	60.12 ± 0.32^eA^	31.02 ± 0.07^dB^	3.72 ± 0.04^dD^	0.00 ± 0.00^cE^	0.00 ± 0.00^bE^	5.03 ± 0.22^aC^
F-EFBB	0.5%	97.35 ± 0.92^cA^	41.77 ± 0.31^cB^	5.65 ± 0.13^bC^	0.82 ± 0.69^bDE^	0.00 ± 0.00^bE^	2.20 ± 0.10^abD^
	1%	68.33 ± 0.64^dA^	28.00 ± 0.05^eB^	1.60 ± 0.08^eC^	0.00 ± 0.00^cD^	0.05 ± 0.05^bD^	0.00 ± 0.00^bD^

**Table 6.** Amount of Pb (µg) in the leachate of contaminated soil amended with EFBB over leaching cycles. Means with the same small letter in the same column are not significantly different. Means with the same capital letter in the same row are not significantly different.

		amount of leachable Pb (µg) in different leachates					
size	rate	1	2	3	4	5	6
control	0%	502.85 ± 5.51^aA^	280.71 ± 2.47^aB^	41.73 ± 0.46^aC^	33.02 ± 1.16^aC^	5.88 ± 0.33^aD^	13.10 ± 0.84^aD^
C-EFBB	0.5%	397.00 ± 2.22^bA^	235.62 ± 0.43^bB^	16.20 ± 0.58^bC^	2.68 ± 0.26^bD^	3.32 ± 0.16^bD^	5.93 ± 0.59^bD^
	1%	230.73 ± 1.68^dA^	111.65 ± 0.60^cB^	6.20 ± 1.02^dC^	1.30 ± 0.12^bD^	0.15 ± 0.15^dD^	0.52 ± 0.52^bD^
F-EFBB	0.5%	295.15 ± 1.59^cA^	110.00 ± 0.20^cB^	11.85 ± 0.41^cC^	0.98 ± 0.09^bE^	1.32 ± 0.45^dE^	5.83 ± 0.34^cD^
	1%	136.77 ± 1.21^eA^	73.90 ± 1.21^eB^	1.07 ± 0.32^eC^	0.13 ± 0.11^bC^	1.92 ± 0.35^cC^	0.00 ± 0.00^cC^

The control soil had significantly higher amounts of Cd or Pb than the 0.5% and 1% application rates of EFBB. Overall, the leachates from both 1% F-EFBB and C-EFBB had significantly lower amounts of Cd or Pb than those from 0.5%. Generally, the F-EFBB-treated soil had significantly lower amounts of Cd or Pb leached from the contaminated soil than the C-EFBB-treated soil.

**Figure 5.**
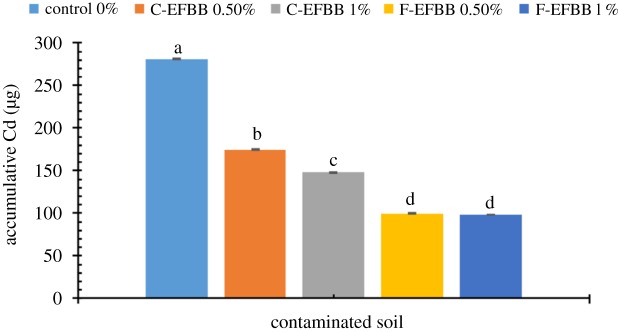
Accumulative amount of Cd in leachate from contaminated soil amended with different particle sizes and rates of biochar (Different letters show significant differences (*p* < 0.05) between the treatment means.)

**Figure 6.**
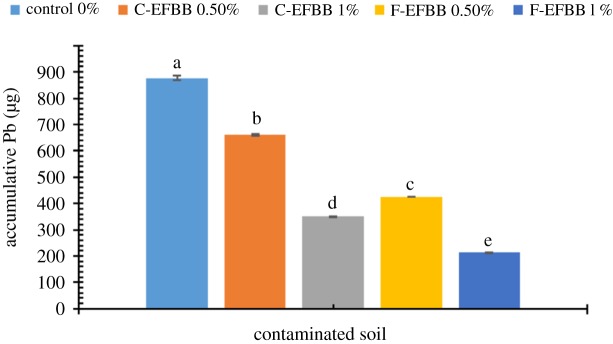
Accumulative amount of Pb leachate from contaminated soil amended with different particle sizes and rates of biochar (Different letters show significant differences (*p* < 0.05) between the treatment means.)

Figures 5 and 6 show the accumulative amount of SRW-leachable Cd and Pb, respectively, from the contaminated soil amended with different particle sizes and rates of biochar. The 1% C-EFBB treatment had a significantly lower accumulative amount of leachable Cd than the 0.5% rate, but both C-EFBB-treated soils had lower accumulative amounts of leachable Cd than the control. However, the accumulative leachable Cd from the 1% F-EFBB-treated soil was not significantly different from the 0.5% F-EFBB-treated soil, but both F-EFBB soils had significantly lower accumulative leachable Cd than the control. At the same application rate, the F-EFBB had a significantly lower accumulative amount of leachable Cd than the C-EFBB.

The 1% application rate of either C-EFBB or F-EFBB had a significantly lower accumulative amount of Pb leached from the soil than the 0.5% application rate, but all the EFBB-treated soil had lower amounts of Pb leached than the control. At a similar application rate, the F-EFBB-treated soil had significantly lower accumulative amounts of Pb leached from the soil than the C-EFBB-treated soil.

## 4. Discussion

### 4.1. The pH, EC and CEC of the contaminated soil

Increasing biochar application rate was reported to increase soil pH, EC and CEC [33–36]. Biochar has a high ash content with the presence of Ca, Mg, K and Na, which can act as liming agents [37,38]. This is why the soil EC increased with increasing biochar application rate in this study. Crushing of biochar to a smaller particle size increases its surface area and exposes the inner functional groups, which can be negatively charged when ionized. The presence of these negative charges resulted in a greater CEC of the soil [39]. The results of this study are consistent with those of Yang *et al*. [40] who found that the application of fine biochar (less than 0.25 mm) at a 5% rate was more effective in increasing soil pH, EC and CEC than a coarser biochar (less than 1 mm) at a 1% lower rate.

### 4.2. Bioavailability and leaching Cd and Pb

SRW is widely used to simulate contaminant leaching through rainfall [23,24,26,41]. Thus, it was used to leach Cd and Pb from the contaminated soil in the current study to simulate tropical soil conditions. The decrease in extracted heavy metals with increasing incubation time was consistent with the result reported by Claoston [12], who found that the leachate of heavy metals from mine tailing-contaminated soil amended with EFB biochar decreased with increasing incubation time. This result is expected because of the ageing reactions of biochar in the soil [38]. These authors found that the mechanism for heavy metal adsorption was through diffusion into the pores of the biochar with respect to the time and age of the biochar in soil. Similarly, the reduction in extracted heavy metals with increasing incubation time could be due to the oxidation on the biochar surface, which creates more adsorption sites for heavy metals adsorption [42,43]. Fresh biochar has a lower content of oxidized functional groups and CEC than aged biochar [44]. Theoretically, biochar with a high surface area has more adsorption sites when the surface area is oxidized by chemical or microbial reactions. Therefore, F-EFBB, which had the higher surface area (table 1), would have had more adsorption sites than C-EFBB for the adsorption of heavy metals.

The reduction in the amount of extractable or leachable Cd and Pb in soil applied with biochar in comparison with the control can be attributed to several factors, including the increasing pH and CEC of the soil, as shown in the incubation study (table 4). This increase in pH and CEC of the soil increased the metal adsorption because of the increase in adsorption sites [10,23,38,45,46]. Similarly, according to Bashir *et al.* [47], the reduction of heavy metals extracted from contaminated soil after the addition of biochar could be due to the increase in soil pH, which would increase in the immobilization of heavy metals through adsorption and precipitation. Increasing the soil pH can immobilize metals in soil because of several reasons. Firstly, increasing the soil pH of variable-charged soils, such as acidic tropical soils, can increase the negative charges on the soil surface, thus increasing metal adsorption [48,49]. Increasing the soil pH leads to the increase in negative charges in soil because of the dissociation of H^+^ from the acidic functional groups of organic matter and clay minerals [48,50–52]. Secondly, the increase in soil pH increases the hydrolysis of heavy metals and, in turn, increases their adsorption by the variable-charged soils because of the higher adsorption affinity by the soil surface for hydroxides metals than the unhydrolysed metal ions [53,54]. Thirdly, increasing the soil pH can affect the precipitation of heavy metals [48,55]. The minimum pH ranges for the precipitation of Cd and Pb hydroxides in the soil system were 8.8–9.8 and 6.1–9.1 for Cd and Pb, respectively [48]. However, the pH range of the soil used in this study was 4.237–4.667 (table 4). Thus, precipitation could be ruled out as the dominant mechanism for Cd and Pb immobilization. The results of this study agreed with the findings of Shen *et al*. [16] who found that it was difficult for biochar to precipitate Pb under acidic environment (kaolinitic soil). Increasing soil CEC had a direct effect on increasing the adsorption of heavy metals [51]. Several studies found that the adsorption capacity of heavy metals such as Pb had a significant correlation with soil CEC [56,57]. Moreover, a higher pH promotes the adsorption of metals on biochar, as the density of the negative charge also increases on the biochar surface [58]. Inyang *et al.* [59] reported that the CEC of plant material biochar was controlled by its functional groups content. Heavy metals could be complexed with biochar functional groups. Stumm and Morgan [60], and Uchimiya *et al.* [41] found that the retention of heavy metals in soil by surface ligands was strongly pH-dependent. However, several researchers have stated that the heavy metals adsorbed by biochars through cation exchange mechanism are readily bioavailable for plants, while those adsorbed by biochar surface functional groups via complexation mechanism are nonbioavailable for plants [61,62]. Shen *et al.* [63] studied the extractability of heavy metals in contaminated soils applied with British broad leaf hard wood biochar pyrolysed at 0.5% and 2% over 3 years and they found that the biochar immobilized heavy metals in the contaminated soils in the long term. However, plant seeds failed to germinate in the contaminated soils regardless of whether they were treated with biochar or not. They suggested a higher application rate of biochar (5% or more) for their effect to be significant. Another study found that biochar amendment on soil contaminated with heavy metals increased the exchangeable fraction of heavy metals after 14 days of incubation compared to the control treatment [64]. Therefore, a field study is required to confirm whether heavy metals in contaminated soils are available for plant uptake after EFBB application.

F-EFBB was more efficient than the coarser biochar in reducing the extractable and leachable Cd and Pb in contaminated soil. This finding can be attributed to the more favourable physiochemical properties of the F-EFBB (higher BET surface area, pH, CEC, negative charge on the surface and the lowest PZC value). Uchimiya *et al.* [41] found that biochar had high oxygen-containing functional groups, which are expected to be more effective in retaining heavy metals, especially in the highly weathered soils. Yang *et al.* [65] indicated that rice straw was more effective than bamboo biochar because of its higher surface area, functional groups and CEC, which had a greater effect on the immobilization of heavy metals in contaminated soil. The results of this study, which showed the finer biochar to be more effective in reducing extractable and leachable Cd and Pb than the coarser biochar, are in agreement with that of Yang *et al.* [65]. The authors found that the fine (less than 0.25 mm) rice straw biochar was more efficient in reducing the availability of heavy metals than the one with the coarse size (less than 1 mm) at the same application rate. They attributed the difference to the higher surface area of the fine biochar. On the contrary, Shen *et al.* [16] found that particle size had no significant effect on the immobilization of Pb in contaminated soil. However, they used biochars equal to or less than 0.15 and 2 mm with a 1% application rate in their study, and the sizes they used might not have been fine enough to expose the inner pores and their functional groups compared with biochars with a fine size of less than 50 µm used in this study.

The reduction in extractable and leachable Cd and Pb from contaminated soil decreased with the increasing application rate of EFBB. The higher application rate increased the pH and CEC of the soil more significantly than the lower application rate (table 4). Similar findings were reported by Claoston [12], who found that the heavy metal immobilization in mine tailings increased with the increase in the rate of EFBB applied. Zhang *et al.* [46] also found an increased heavy metal immobilization with an increase in biochar application rate, and they attributed this result to the increase in soil pH.

Shen *et al.* [64] investigated the stability of heavy metals with soil washing residue amended with biochar and they suggested there was a risk associated with the process which could be due to the presence of fine soil particles in the soil washing residue. However, in this study, we did not observe any biochar with fine particles in the leachates. However, the potential of long-term stability of heavy metals in contaminated soils amended with different particle sizes of EFBBs needs to be investigated, especially the possible risk from the exchangeable fractions.

## 5. Conclusion

The results revealed the potential of C-EFBB and F-EFBB in improving selected soil properties and in reducing the SRW-extractable and -leachable Cd and Pb from contaminated soil. The addition of C-EFBB and F-EFBB increased the pH, EC and CEC of the contaminated soil. The amounts of SRW-extractable and -leachable Cd and Pb significantly decreased with the EFBB application. The lowest extractable and leachable Cd and Pb were observed from 1% F-EFBB-treated soil. The amount of extractable and leachable Cd and Pb decreased with increasing incubation time and leaching cycles. The application of F-EFBB to Cd- and Pb-contaminated soil can immobilize the heavy metals more than that of C-EFBB. Therefore, the use of EFBB can be recommended for the remediation of heavy metal-contaminated soils, and biochar with a fine particle size can reduce the application rates more than the coarser biochar. However, long-term studies on the phytoavailability of Cd and Pb are necessary to confirm the findings.

## Data accessibility

All datasets supporting this article have been located within the manuscript.
